# Low-threshold potassium currents stabilize IID-sensitivity in the inferior colliculus

**DOI:** 10.3389/fncir.2012.00060

**Published:** 2012-08-31

**Authors:** Anita Karcz, Rudolf Rübsamen, Cornelia Kopp-Scheinpflug

**Affiliations:** ^1^Carl-Ludwig-Institute for Physiology, University of Leipzig, Medical SchoolLeipzig, Germany; ^2^Faculty of Biosciences, Pharmacy and Psychology, University of LeipzigLeipzig, Germany; ^3^Medical Research Council UK, Toxicology UnitLeicester, UK

**Keywords:** potassium channels, IID processing, temporal precision, sound localization

## Abstract

The inferior colliculus (IC) is a midbrain nucleus that exhibits sensitivity to differences in interaural time and intensity (ITDs and IIDs) and integrates information from the auditory brainstem to provide an unambiguous representation of sound location across the azimuth. Further upstream, in the lateral superior olive (LSO), absence of low-threshold potassium currents in *Kcna1*^−/−^ mice interfered with response onset timing and restricted IID-sensitivity to the hemifield of the excitatory ear. Assuming the IID-sensitivity in the IC to be at least partly inherited from LSO neurons, the IC IID-encoding was compared between wild-type (*Kcna1*^+/+^) and *Kcna1*^−/−^ mice. We asked whether the effect observed in the *Kcna1*^−/−^ LSO is (1) simply propagated into the IC, (2) is enhanced and amplified or, (3) alternatively, is compensated and so no longer detectable. Our results show that general IC response properties as well as the distribution of IID-functions were comparable in *Kcna1*^−/−^ and *Kcna1*^+/+^ mice. In agreement with the literature IC neurons exhibited a higher level-invariance of IID-sensitivity compared to LSO neurons. However, manipulating the timing between the inputs of the two ears caused significantly larger shifts of IID-sensitivity in *Kcna1*^−/−^ mice, whereas in the wild-type IC the IID functions were stable and less sensitive to changes of the temporal relationship between the binaural inputs. We conclude that the IC not only inherits IID-sensitivity from the LSO, but that the convergence with other, non-olivary inputs in the wild-type IC acts to quality-control, consolidate, and stabilize IID representation; this necessary integration of inputs is impaired in the absence of the low-threshold potassium currents mediated by Kv1.1.

## Introduction

Interaural intensity differences (IIDs) are essential cues to localize the source of high-frequency sounds. Individual IID-sensitive neurons in the auditory brainstem lateral superior olive (LSO) and in the auditory midbrain inferior colliculus (IC) integrate excitation received from one ear with inhibition received from the other ear, a computation that results in a specified firing rate for each IID (Boudreau and Tsuchitani, 1968; Sanes and Rubel, 1988; Tollin, 2003). The response rate of an individual neuron is tuned to a particular IID range. On a population level, this will result in IID functions that represent the whole range of physiological relevant IIDs. While the encoding of IID functions is quite well agreed in the LSO, in the IC there is some debate as to whether IID-sensitivity is generated de novo or is inherited from the upstream LSO (Park, 1998; Pollak et al., 2003; Gittelman et al., 2009). Although neurons in the IC receive strong excitatory projections from the contralateral LSO carrying information about IID-sensitivity, there are additional excitatory inputs from the contralateral VCN (Beyerl, 1978; Moore and Kitzes, 1985; Ross et al., 1988) and bilateral inhibitory inputs from the dorsal nucleus of the lateral lemniscus (DNLL) (Shneiderman et al., 1993). These non-olivary inputs create and moreover modify IID-sensitivity in the IC (Pollak et al., 2003).

As suggested by previous studies in the LSO and the IC, the arrival of the binaural inputs into a binaural level-detector within a submillisecond range of each other is crucial for their integration (Irvine et al., 1995; Joris and Yin, 1995). A delay in one input will result in a shift of the respective IID-function. Different models exist for the generation of positive (excitatory ear more intense) or negative (inhibitory ear more intense) IIDs, requiring the inputs to arrive either with a constant delay or to be coincident (Park et al., 1996). The time window for integrating synaptic inputs depends strongly on the neuronal membrane time constant and therefore on the ion channels that are open at a given membrane potential. Kv1.1 containing channels are activated at depolarizations very close to the resting membrane potential. They repolarize the membrane quickly and are therefore powerful means of restricting temporal summation (Oertel, 1985; Manis and Marx, 1991; Trussell, 1999; Johnston et al., 2010). In the LSO, the lack of Kv1.1 caused a limited IID representation with IIDs corresponding only to sounds in the ipsilateral hemifield (Karcz et al., 2011). Here we aim to understand how a mismatched timing of the inputs into the IC alters the neurons' IID-sensitivity and how the presence of low-threshold potassium channel subunits like Kv1.1, prevents a disrupting influence of poorly-timed inputs. Using Kv1.1 knockout mice as a model allows us to study not only the effect of a shift in stimulus onset timing on IID encoding (as can be achieved also by the introduction of a stimulus delay) but also the effect of altered intrinsic excitability on the generation of each action potential that will be integrated to an IID response. We use single-unit recording *in vivo* from wild-type mice (*Kcna1*^+/+^ mice) and also from mice whose temporal integration window is artificially extended through the lack of Kv1.1 containing channels (*Kcna1*^−/−^ mice).

## Methods

A detailed description of the methods and mouse strains used in this study was described previously in (Karcz et al., 2011). In brief, the *Kcna1*^*tm*1*Tem*^ strain was generated as described in Smart et al. (1998). Mice used in this study were generated by intercrossing heterozygotes from the C3HeB/FeJ-*Kcna1*^*tm*1*Tem*^ line maintained at the University of Leipzig. The experiments were performed at the Neurobiology Laboratories of the Faculty of Bioscience, Pharmacy and Psychology of the University of Leipzig (Germany). All experimental procedures were approved by the Saxonian District Government, Leipzig, and were conducted according to European Communities Council Directive of 24th November 1986 (86/609/EEC). Genotyping was carried out on DNA isolated from tail clips of each mouse in a litter aged 7–10 days as described by Brew et al. (2003). Detailed protocols are available online (http://depts.washington.edu/tempelab/Protocols/KCNA1.html).

### Surgical preparation

During the experiments and surgical preparation, the animals were anaesthetized with a combined initial dose of 0.01 ml/g body weight of ketamine hydrochloride (100 mg/kg body weight; Parke-Davis, Berlin, Germany) and xylazine hydrochloride (5 mg/kg body weight; Bayer, Leverkusen, Germany). Anaesthesia was maintained throughout the recording experiments by hourly injections of one-third of the above dose. Ketamine is an antagonist of NMDA receptors. Ketamine-Xylazine anesthesia was employed in previous studies using the same mouse strain (Kopp-Scheinpflug et al., 2003; Karcz et al., 2011) and no differences in the depth or duration of anesthesia were found between genotypes. Other anesthetics like Isoflurane are less suited for the present experiments since it inhibits hyperpolarization-activated cyclic nucleotide modulated channels (Chen et al., 2009) which have a close structural resemblance to voltage-gated potassium channels. To avoid infections all instruments were wiped with a 70% alcohol before surgery. Recording and tracer solutions were filtered and electrodes were heat-sterilized. The incision site on the experimental animals head was cleaned with a chlorhexidine solution and the skull was exposed along the dorsal midsagittal line. A small metal bolt for supporting the animal in the stereotaxic recording device was glued to the bone overlaying the forebrain. Two holes were drilled in the skull, one to position the reference electrode in the superficial cerebellum, the other for insertion of recording electrodes. The second drill hole (500 μm diameter) was located 1.2 mm lateral to the midline and 1.8 mm caudal to the lambda suture. All recording experiments were performed in a sound-attenuated chamber (Industrial Acoustics, Type 400). During surgery and experiments the body temperature was kept between 36°C and 37.5°C by positioning the animal on a temperature-controlled heating pad (Harvard Apparatus) and maintaining the temperature of the sound-attenuated chamber at 25–30°C. Warmed lactated Ringer's (0.5–1 ml) was injected subcutaneously to prevent dehydration. At the end of the experiment, the head post was removed under anesthesia and a local analgesia (Xylocain Gel 2%; AstraZeneca GmbH) was applied to the wound, before closing the incision with histoacrylic glue. To help recovery from anesthesia mice were placed in oxygen-enriched air. Food pellets soaked in water were given to the mice for additional fluid supply. Over the duration of 4–5 days the animals were monitored daily for bodyweight, abnormal behavior or signs of infections.

### Data collection

Recordings were made using glass micropipettes (15–30 MΩ, 3 M KCl). The electrode was advanced vertically by a motorized micromanipulator (WPI, DC3001). The activity of isolated single units was identified by their relatively constant spike height, filtered (0.3–10 kHz), amplified (TDT, PC1) to the voltage range of the spike discriminator (TDT, SD1), and recorded at 100 kHz using custom-written software (Dr. M. Weick, University of Leipzig).

### Acoustic stimulation

Stimulus tones (100/40 ms duration, 5 ms rise-fall time, 100–300 ms inter-stimulus interval) were generated at a 97.7 kHz sampling rate using (TDT; RP2-1), amplified (TDT; ED1), delivered to electrostatic speakers (TDT; EC1) and fed via acoustic tubing to the outer ear approximately 5 mm from the animal's eardrum. For each neuron the following stimulation protocol was employed.

Excitatory response maps were measured by random presentation of pure-tone pulses (100 ms duration, 5 ms rise-fall time, 100 ms interstimulus interval) within a given matrix of 16 × 15 frequency/intensity pairs (240 combinations). The frequency range of this matrix typically included 5 octaves (4 tones/octave) with about three octaves below the estimated characteristic frequency (CF) and two octaves above. The intensity varied in 5 dB-steps between 90 and 20 dB SPL. Each frequency/intensity combination was presented five times in a predefined frequency/intensity array (1200 tone bursts). The timing and number of spikes were measured during the 100 ms period of stimulus presentation. Spontaneous activity was defined by the firing rate during eighty 100 ms-periods (8 s) with maximal attenuation (120 dB) of the stimulus randomly inserted in the stimulus protocol. Excitatory response areas were defined by the range of frequency/intensity combinations that evoked firing rates at or above the 10% significance level above spontaneous activity (Dorrscheidt, 1981). The outline of the response area defined the unit's frequency-threshold curve, from which the CF and the threshold at CF were determined. Vice versa, inhibitory response areas were defined by a respective reduction of firing rates below the level of spontaneous rate (or below an acoustically evoked firing rate; for details see Karcz et al., 2011). Rate-level functions were calculated at CF by averaging the spike rates for each stimulus intensity (5 repetitions/stimulus). Median first spike latency and jitter (25–75% range of first spike latencies) were determined from 250 stimulus repetitions at CF and 80 dB SPL. Temporal response patterns were evaluated from peri-stimulus time histograms that display the number of APs occurring within 0.5 ms time intervals during stimulus presentation. Sensitivity to IIDs was tested by presenting a constant tone (40 ms) at CF, 20 dB (±15 dB) above threshold to the contralateral ear (using these parameters, similar firing rates could be achieved in both genotypes) and simultaneously stimulating the ipsilateral ear at CF with stimulus intensities pseudo-randomly varying from 20 to 90 dB SPL in 5 dB-steps. The acquired averaged IID functions (25 repetitions) were normalized; with AP firing rates during monaural contralateral stimulation corresponding to 100%. For further quantification of IID_50_ and slopes, IID functions were fit with four-parametric sigmoid functions from which IID values at 50% reduction of firing rate (IID_50_) were determined (Wise and Irvine, 1985). Data were only considered if the fit had a minimum of 95% correlation.

### Statistical analyses

Statistical analyses of the data were performed with SigmaPlot™ (Version 12; Systat Software Inc., San Jose, CA, USA). Unless indicated otherwise, results are expressed as mean ± standard error of the mean (S.E.M.) or for non-normal distributions as median (25%; 75% quartiles). Statistical significance was assessed using two-tailed tests; the Student's *t*-test for normally distributed data and the Mann-Whitney Rank Sum Test for non-normal distributions. Normality was assessed by the Shapiro-Wilk test. Significance levels are indicated by p-values that were corrected for nonhomogeneity of between cell-correlations by the Hunyh-Feldt method.

### Verification of recording sites

In each animal, all recording sites were verified histologically by iontophoretic injections (2 μA for 5 min) of hydroxystilbamidine (FG; Biotium; equivalent to FluoroGold™) at the end of the recording session. The mice were allowed to recover from anaesthesia and after 5 days (FG) the animals received a lethal anaesthetic injection and were then perfused via the left ventricle with 0.9% NaCl solution followed by fixative (2.5% paraformaldehyde in 0.1 M phosphate buffer, pH 7.4) for 20–25 min. The brains were sectioned on a vibratome and the tissue sections (30 μm thick) were examined using a fluorescence microscope (Zeiss, Axioskop, absorption/emission: 361/536 nm) and the electrode tracks and recording sites were identified.

## Results

This study is based on single-unit recordings of IID sensitive neurons in the IC, which receive contralateral excitation and ipsilateral inhibition. A sample of 19 wild-type (5 *Kcna1*^+/+^ mice) and 19 knockout (5 *Kcna1*^−/−^ mice, average age: 39 days) IC neurons was recorded and the neurons' basic response features assessed by their response to the excitatory ear. If the excitatory input conveys mainly inherited information from the LSO, the basic response features may not differ any more than in their input structure. Indeed, characteristic frequencies of 14.8 ± 1.1 kHz (*Kcna1*^+/+^) and of 17.7 ± 2.2 kHz (*Kcna1*^−/−^) did not differ between genotypes (*p* = 0.247). Spontaneous firing rates were low in both, *Kcna1*^+/+^ mice (0.2 [0.0; 3.0] spikes/s) and *Kcna1*^−/−^ mice (0.5 [0.0; 5.4] spikes/s; *p* = 0.460) and despite a 18 dB threshold elevation (*Kcna1*^+/+^: 38 ± 3 dB SPL; *Kcna1*^−/−^: 56 ± 4; *p* = 0.002), evoked firing rates at 20 dB above threshold did not differ significantly between both genotypes (*Kcna1*^+/+^: 40 [18; 106] spikes/s; *Kcna1*^−/−^: 16 [12; 32] spikes/s; *p* = 0.072; Table 1). The elevation of thresholds by 18 dB in *Kcna1*^−/−^ IC neurons is similar to the threshold elevation found in the upstream LSO (Karcz et al., 2011). No further significant threshold elevation has been detected between IC neurons of *Kcna1*^+/+^ nor of *Kcna1*^−/−^ mice (Two Way ANOVA, interaction genotype vs. nucleus, *F*_(1, 93)_ = 1.87, *p* = 0.175, η^2^_p_ = 0.02). First spike latencies and their variability (jitter) as well as temporal response patterns were used to compare the timing precision in *Kcna1*^+/+^ and *Kcna1*^−/−^ neurons. Consistent with the changes found in the LSO of *Kcna1*^−/−^ mice (Karcz et al., 2011) no significant genotype specific differences were found in latencies and jitter (Table 1). Interestingly, the distribution of temporal response patterns changed from chopper-PSTHs (dominant in wild-type) to more onset-PSTHs in the *Kcna1*^−/−^ neurons, indicative for a change in the IC neurons' intrinsic response properties. The classification of the PSTHs was based on the definitions by Pfeiffer (1966). Chopper PSTHs exhibit several regularly spaced peaks with inter-peak distances unrelated to the stimulation frequency. In the present study, chopper units were not further divided into transient choppers which display the regular discharge pattern only at the beginning of the response, and in sustained choppers where prominent periodic discharge peaks were found throughout the entire duration of the response. Units classified as firing onset responses have PST histograms that are characterized by one or two initial peaks with little activity thereafter.

**Table 1:** **Response features of IID-sensitive IC neurons**.

**Parameter**	***Kcnal*^+/+^**					***Kcnal*^−/−^**					**Test**	**Significance**
	**Mean ± S.E.M.**	**Median**	**25%**	**75%**	***n***	**Mean ± S.E.M.**	**Median**	**25%**	**75%**	***n***		
CF_exc_ (kHz)	14.8 ± 1.1	13.8	11.3	17.8	19	17.7 ± 2.2	13.8	11.0	24.4	19	*t*-test	*p* = 0.247
CF_inh_ (kHz)	19.3 ± 2.0	19.7	13.3	24.3	17	17.5 ± 2.8	13.8	11.5	22.9	11	MWRS-test	*p* = 0.495
Threshold_exc_ (dB SPL)	38 ± 3	40	26	53	19	56 ± 4	63	50	69	19	*t*-test	*p* = 0.002
Threshold_inh_ (dB SPL)	46 ± 5	45	35	65	17	61 ± 6	60	46	82	11	*t*-test	*p* = 0.089
Spont. rate (spikes/s)	2.3 ± 0.9	0.2	0.0	3.0	19	5.0 ± 2.2	0.5	0.0	5.4	19	MWRS-test	*p* = 0.460
Firing rate (spikes/s)	57 ± 10	40	18	106	19	38 ± 11	16	12	32	19	MWRS-test	*p* = 0.072
20 dB>thres.												
Latency (ms)	12.0 ± 2.1	6.7	5.7	19.7	13	7.5 ± 0.7	6.5	5.5	8.7	18	*t*-test	*p* = 0.238
Jitter (ms)	1.7 ± 0.4	1.4	0.3	3.0	11	0.8 ± 0.3	0.4	0.2	0.8	18	MWRS-test	*p* = 0.078
PSTH (%)	Chopper	55			6/11	Chopper	12			2/17		
	Onset	45			5/11	Onset	65			11/17		
	Primary like	0			0/11	Primary like	18			3/17		
	Other	0			0/11	Other	6			1/17		
11D_50_ (dB)80 dB	7.6 ± 3.1	10.5	0.9	13.8	7	7.2 ± 3.8	8.4	−2.0	15.5	7	*t*-test	*p* = 0.938
IlDslope 80 dB	4.8 ± 1.0	4.4	3.4	6.1	7	4.2 ± 0.8	4.2	2.6	6.0	7	*t*-test	*p* = 0.645
11D_50_ (dB)20 dB > thres.	8.5 ± 4.8	10.7	0.5	22.8	11	8.5 ± 3.7	8.7	−0.3	16.1	8	*t*-test	*p* = 0.992
I I Dslope20 dB > thres.	3.9 ± 1.0	2.8	1.6	6.6	11	5.0 ± 1.4	4.3	1.8	6.2	8	MWRS-test	*p* = 0.536

MWRS-test: Mann–Whitney Rank sum-test.

### IID-sensitivity in IC neurons covers the whole range of physiological relevant IIDs

**Figure 1:**
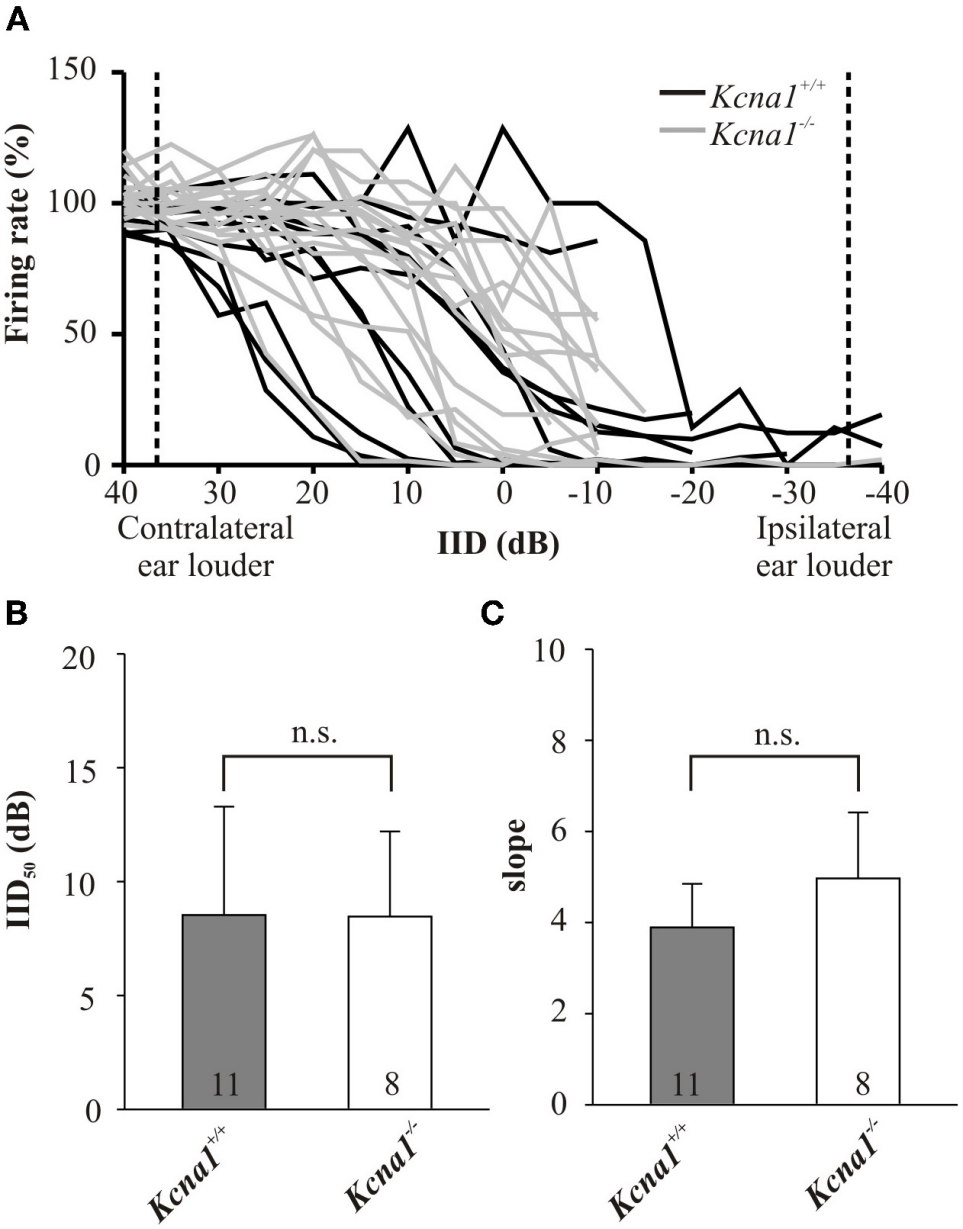
**(A–C)** IID-sensitivity of IC neurons in wild-type and *Kcna1*^−/−^ mice. **(A)** IID functions obtained from the IC of *Kcna1*^+/+^ (black curves) and *Kcna1*^−/−^ mice (gray curves) are equally distributed between negative and positive IIDs. Dotted lines represent the range of IIDs which a small headed species such as the mouse could possibly encounter in a natural environment (Chen et al., 1995). **(B)** IID curves were fitted by a 4-parametric sigmoid function from which IID values corresponding to a 50% rate reduction (IID_50_) were determined. No genotype specific differences were found. **(C)** The slope of the IID functions (determined from the sigmoid fit) was also not significantly different between the two genotypes.

**Figure 2:**
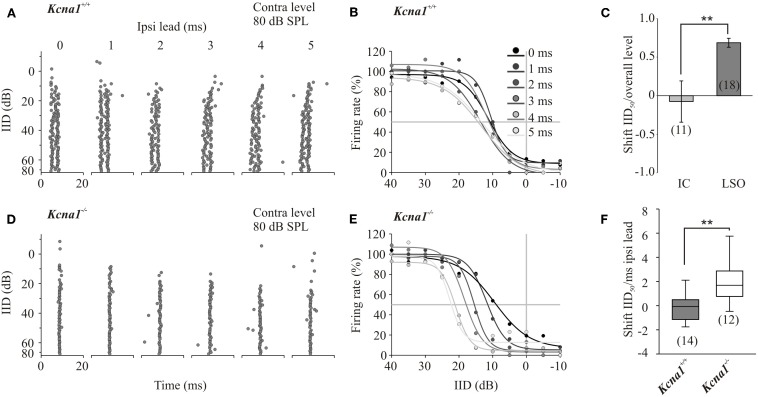
**(A–F)** Increasing temporal mismatch between inputs shifts IID functions in the *Kcna1*^−/−^ but not the wild-type mice. Instead of the simultaneous onset of the ipsi- and contralateral stimulus, the ipsilateral inhibitory stimulus was presented 1–5 ms in advance. **(A)** Dot raster displays of a representative *Kcna1*^+/+^ neuron show no systematic change in response to different ipsilateral lead times. **(B)** IID functions of the same neuron shown in A did not vary in their IID_50_ values. **(C)** IID-sensitivity in wild-type IC neurons was not only stable toward temporal mismatch in the inputs; they also showed a larger stability toward overall changes in sound intensity compared to wild-type LSO neurons. **(D)** Increasing the delay between excitatory and inhibitory inputs caused increasingly more effective inhibition in *Kcna1*^−/−^ neurons generating a remarkable shift of IID sensitivity toward more positive IIDs **(E)**. This shift in IID-functions with temporal lead of the inhibitory input is quantified in panel **(F)**.

IID sensitivity between *Kcna1*^+/+^ and *Kcna1*^−/−^ IC neurons was compared based on the distribution of IID functions within the range of physiological relevant IIDs quantified by IID_50_ values (Figures 1A,B). The neurons' firing rates at sole stimulation of the excitatory (contralateral) ear was normalized and set to 100%. As differences in overall sound intensity will lead to a shift in IID-sensitivity at least in the LSO (Tsai et al., 2010; Karcz et al., 2011), the distribution of IID 50 values was evaluated for two conditions: (1) same excitatory intensity of 80 dB SPL for all neurons and (2) 20 dB above each unit's individual excitatory threshold. For both conditions, successively increasing the intensity at the inhibitory (ipsilateral) ear eventually reduced the firing rate to at least 50% (= IID_50_). Neither condition showed genotype-specific differences, suggesting that the lack of Kv1.1 mediated currents did not change the distribution of IIDs on the IC population level (Figures 1B,C and Table 1). Analyses on the single neuron level in wild-type mice confirmed a lower sensitivity to overall sound intensity as has been reported for the IC compared to the LSO (Park et al., 2004): the shift of IID sensitivity elicited in *Kcna1*^+/+^ IC neurons did not differ significantly from zero (one-sample *t*-test: *p* = 0.782) whereas level-dependence of IID responses was present in LSO neurons with shifts toward positive IIDs of 0.68 dB per dB excitatory level increase (shift IID_50_, MWRS-test, *p* = 0.023; IC^+/+^: −0.30 [−0.41; 0.70] dB/dB; LSO^+/+^: 0.68 [0.48; 0.87] dB/dB; Figure 2C).

### In the IC, low-threshold potassium currents prevent integration of inputs raised by non-physiological IIDs

One function of the IC in IID processing is thought to be an additional modification of IID-sensitivity inherited from the LSO. It has already been suggested that IID-sensitivity in IC neurons is less sensitive toward changes in overall sound intensity (Park et al., 2004; Tsai et al., 2010). Here we explore the role of Kv1.1 low-threshold potassium currents in temporal integration of synaptic inputs into the IC. Therefore, the main excitatory input from the contralateral LSO remained unaltered and a temporal lead of 1, 2, 3, 4 or 5 ms to the stimulus at the ipsilateral ear was introduced. Temporally mismatched, additional non-olivary inputs will have a larger influence on the IID-sensitivity in the IC of *Kcna1*^−/−^ mice as their integration window should be larger. The shift of IID_50_ values per temporal lead was calculated for each neuron including all IID functions that show 95% correlation with 4-parametric sigmoidal fits (Figure 2). Positive values represent shifts toward more positive IIDs and indicate more effective inhibition present at lower ipsilateral intensities of the temporally advanced stimulus. Accordingly, a negative shift in IID_50_ values per millisecond temporal lead displays the need for higher ipsilateral inhibitory intensities to suppress 50% of the contralaterally evoked firing rate. The initial contralateral firing rate was evoked by either stimulation at the neuron's CF and 80 dB SPL or at 20 dB above the neuron's threshold. Under both conditions, the introduction of a temporal lead of the inhibitory signal caused the IID functions of *Kcna1*^−/−^ IC neurons to shift to more positive IIDs, whereas no consistent shift was detected in *Kcna1*^+/+^ neurons (contra-intensity 80 dB: shift IID_50_, *Kcna1*^+/+^: 0.1 ± 0.5 dB/ms; *Kcna1*^−/−^: 1.9 ± 0.6 dB/ms, *p* = 0.03; contra-intensity 20 dB above threshold: *Kcna1*^+/+^: −0.3 −0.5; −0.1] dB/ms; *Kcna1*^−/−^: 1.5 [0.7; 2.6] dB/ms, *p* = 0.005; Figure 2F). The effects are illustrated for a representative neuron of each genotype in Figure 2. The dot raster plots display the occurrence of an AP at a certain time point for five repetitions of each of the 16 tested IIDs (Figures 2A,D). In the wild-type example, different lead times did not systematically change IID sensitivity. The same data plotted in form of IID functions (Figures 2B,E) summarizes the weak influence of temporal lead on IID_50_ values in this *Kcna1*^+/+^ neuron (shift of IID_50_: 0.5 dB/ms). In contrast, the inhibitory effect in the *Kcna1*^−/−^ neuron presented in Figure 2 consistently increased with increasing ipsilateral inhibitory lead time, resulting in a shift of IID_50_ values of 3.5 dB/ms. On average, *Kcna1*^−/−^ IC neurons have an increased susceptibility toward changes in the arrival times of the binaural inputs, resulting in IID_50_ shifts even outside the range of physiological relevant IIDs (Figure 2F) where they would no longer add reliable information about the location of a sound source. In wild-type animals, the presence of Kv1.1 currents restricts the integration of such a temporal mismatch between LSO and non-olivary inputs and stabilizes the IID functions within the physiological range.

## Discussion

The presence of low-threshold potassium currents (Kv1.1) is known to limit action potential firing and restrict temporal summation of synaptic inputs. In the present study we used the *Kcna1*^−/−^ mouse as a model to evaluate the impact of changes in the integration time on IID-sensitivity in the IC. Our results suggest that although a major part of the IID-sensitivity reflects the input from the LSO, correct additional integration of other, non-olivary inputs is necessary to stabilize IID-sensitivity against variations in input timing.

### Kv1.1 currents control the neurons' synaptic and intrinsic firing pattern

Kv1.1 containing channels activate at membrane potentials around −40 mV which is only slightly positive to the neurons' resting membrane potential (Brew and Forsythe, 1995). As sodium currents are activated in a similar voltage range, Kv1.1 mediated currents and sodium currents compete, pulling the membrane potential in hyperpolarizing and depolarizing directions, respectively. Therefore, only strong and fast inputs, as found in cells with coincident inputs or in cells with giant calyceal input, activate sodium currents quickly enough to overcome the competition with Kv1.1 (Oertel, 1985; Manis and Marx, 1991; Trussell, 1999; Johnston et al., 2010). The resulting brief synaptic integration window will restrict temporal summation of synaptic inputs, excitatory and/or inhibitory, to a narrow coincidence window. Vice versa, the lack of Kv1.1 containing channels will allow temporal integration of more disperse inputs, which—in the present study—will lead to a less stable encoding of IID-sensitivity in the IC.

Besides restricting synaptic integration, Kv1.1 mediated currents also determine the intrinsic firing pattern of the neurons. These currents repolarize the membrane quickly and inactivate slowly, which makes firing of subsequent action potentials highly unlikely. Accordingly, Kv1.1 mediated currents are strongly associated with single-spike firing in response to prolonged depolarizations in patch-clamp studies from brain slices (Brew and Forsythe, 1995; Barnes-Davies et al., 2004; Cao et al., 2007). Although it appears as an obvious assumption, this single-spike firing phenotype does not necessarily correspond to single spike (onset) firing in responses to sound burst stimulation *in vivo*. In contrast to a prolonged depolarization by current injection *in vitro*, sound-evoked synaptic transmission is composed of many discrete transient events greatly varying in temporal succession and amplitudes and thus Kv1.1 mediated currents will foster single spiking to EPSP depolarizations at each of these events. Hence, the stronger expression of Kv1.1 will promote single spike responses to a sustained *in vitro* depolarization AND will at the same time enhance discrete firing as in chopper/tonic responses, *in vivo*. Thus, the temporal firing patterns change from wild-type to *Kcna1*^−/−^ mice from single-spiking to multiple firing in the slice and from chopper/Primary-like (tonic) to onset (phasic) discharge patterns *in vivo*. Respective changes were observed in the probability of temporal firing patterns from chopper to onset in the LSO and the IC of *Kcna1*^−/−^ mice (Table 1; Karcz, 2011). The prevalence of chopper responses in the wild-type LSO and IC suggests that this is the optimal response pattern for integrating temporally precise binaural inputs over several cycles of the stimulus, whereas in the *Kcna1*^−/−^ mouse the respective information is restricted to the response onset.

### Origin of IID sensitivity in IC neurons

One difficulty in the evaluation of the activity in IC neurons is the lack of knowledge about the origin of binaural response patterns. It is likely that the recorded cell population resembles a mixture of IC neurons that either (1) inherit their IID sensitivity unmodified from neurons in the contralateral LSO, (2) inherit some IID sensitivity from neurons in the contralateral LSO which is modified in the IC by additional inputs from non-olivary nuclei, or (3) generate IID sensitivity *de novo* by receiving inputs from non-olivary nuclei converging at the level of the IC (for review see Pollak et al., 2002). The latter two options require precise temporal integration as secured by Kv1.1. One possible source assumed to play a role in shaping or in *de novo* generation of IID sensitivity in IC neurons is the DNLL. This nucleus is innervated by excitatory contralateral CN neurons as well as by ipsilateral and contralateral LSO neurons. DNLL principal neurons send inhibitory GABAergic projections to both ICs (Adams and Mugnaini, 1984; Kelly et al., 2009). Pollak et al. (2002) reported four differential effects on IE neurons of the IC, when GABAergic inhibition originating in the DNLL was blocked (Pollak et al., 2002). Most common was a shift of the IID function to more negative IID values. Hence, a more intense inhibitory stimulus was necessary to achieve maximal reduction of firing compared to the condition with an effective GABAergic DNLL inhibition. The application of bicuculline, a GABA antagonist, or the inactivation of the DNLL contralateral to the IC with kynurenic acid caused equivalent IID shifts. The authors assumed that IID sensitivity in DNLL and LSO neurons differs slightly in that complete inhibition is achieved in the DNLL whilst LSO neurons would still dynamically integrate excitatory and inhibitory inputs. Those differences in IID sensitivity between DNLL and LSO could result from different latency mismatches but involved circuitries are not yet clarified. Tsai et al. (2010) suggested a subtraction model to demonstrate the larger level invariance of IC compared to LSO neurons shown by Park et al. (2004) which was also confirmed is the present study. The subtraction model suggests that each LSO neuron's IID function has its mirror image IID function in a neuron of the opposite LSO (ipsilateral to the IC). The IC then integrates the excitatory responses conveyed by the contralateral LSO and the inhibitory mirror image responses transferred from the ipsilateral LSO via the contralateral DNLL (for details see Figure 5 in Tsai et al., 2010). On the level of the IC strong excitation would converge with weak inhibition. The interplay of the excitatory and inhibitory counterparts originating from the same source remove the bias in form of shift of IID functions due to overall level changes. Failure to integrate such non-olivary inputs as suggested for the *Kcna1*^−/−^ mice, results in larger vulnerability toward changes in overall level or input timing. Besides the actual arrival time of the two inputs, the temporal jitter of the inputs is another parameter that can disrupt binaural integration (Gittelman and Pollak, 2011). Broadening the temporal jitter of at least one input results in a displacement of the IID-functions in the LSO (Karcz et al., 2011).

Taken together, low-threshold potassium currents act on at least two different levels: I) control of intrinsic spike firing behavior and II) restriction of temporal summation of synaptic inputs. In the IC this results in a shift from chopper to onset response patterns, which can cause a loss of information normally obtained by integrating binaural information during subsequent cycles in the temporal response pattern following complex-structured acoustic signals. So it is conceivable that the contralateral LSO provides the IC neurons with the basic IID information while additional inhibitory inputs from non-olivary nuclei (arriving with a slight delay) are integrated at the level of the IC to remove level variance and to consolidate IID responses of physiologically timed binaural inputs. Inputs arriving outside the IC neurons' integration window might then be attributed to separate sound objects. A disruption of synaptic integration in the IC as in the *Kcna1*^−/−^ mice can then interfere with such quality-control function performed by non-olivary inputs and subsequently result in impaired sound localization in *Kcna1*^−/−^ mice (Allen and Ison, 2012).

### Conflict of interest statement

The authors declare that the research was conducted in the absence of any commercial or financial relationships that could be construed as a potential conflict of interest.
